# First Case Report of a Near Lethal Envenomation by a *Salomonelaps par* (Solomons Coral Snake) in the Solomon Islands

**DOI:** 10.3390/tropicalmed3030090

**Published:** 2018-08-21

**Authors:** Sarah Luthy, Damian Rake, Tanya Buchanan, Christine Schultze

**Affiliations:** 1 GP Obstetrician, Cairns, Queensland 4870, Australia; 2 Ngukurr Clinic, Ngukurr 0852, Northern Territory, Australia; damian.rake@my.jcu.edu.au; 3 Peninsula Private Hospital, Frankston 3199, Victoria, Australia; taneshka@bigpond.com; 4 RN Kantonspital Basel 4031, Switzerland; dc.schultze@bluewin.ch

**Keywords:** snake bite, Solomon Islands, neurotoxicity, *Salomonelaps par*, Solomons coral snake

## Abstract

Venomous snake bites in the Solomon Islands can be very dangerous due to lack of access to health care. There are no documented case reports of envenomation by snake bites in the Solomon Islands. This case report highlights the management of a patient with potentially lethal neurotoxicity secondary to a *Solomonelaps par* (Solomons coral snake) in a low resource setting. This case identifies the potential benefit of further research to determine the incidence of lethal envenomation as well as analysing the venom to determine if any commercially available antivenom would be useful in the treatment of envenomation by *Salomonelaps par* and other venomous snakes. There should be consideration given to providing education on first aid for people living in remote areas as well as education for health workers.

Venomous snake bites in the Solomon Islands can be potentially lethal, as many people live in remote areas with poor access to clinics or hospitals. Hospitals are not equipped with high-dependency or intensive care facilities and no antivenom is available in the country. Up until now, there have been no documented cases of envenomation by snakes in the Solomon Islands, although there have been a few anecdotal reports of fatalities from people living in very remote areas. This is a case study of a patient that was envenomated, paralysed, and survived in a small remote hospital with minimal resources.

A 15-year-old boy presented to a remote hospital in Malaita province in the Solomon Islands after sustaining a snake bite to his leg. The 70-bed hospital was staffed by one doctor, two physician assistant students on work experience, nurses, and health workers.

The patient was bitten three times on the right ankle at around 3 p.m. on a Friday afternoon in a remote bush garden. The snake refused to let go, requiring him to cut it with his machete, which caused a lethal caudal wound on the body of the snake. Sometime after that, he created a tourniquet from a piece of vine and tied it around his leg. After being carried by his family for several hours through the bush, he arrived at the hospital around 7:30 p.m.

Upon arrival, he had marked paralysis (including ptosis) with fasciculations of his arms and legs and was unable to sit unsupported. He was profusely salivating and lathered in sweat. He had large dilated pupils that were contrary to his other cholinergic signs. His vital signs were pulse 60 and blood pressure 150/100. There were irregular gasps with secretions bubbling out of his mouth. His oxygen saturation declined rapidly from 98% to 76% on room air. There were coarse crackles throughout on examination of his respiratory system. His right leg was noticeably swollen, with bite marks evident on the ankle. The swelling was thought to be due to the tourniquet, which by this time had been on for approximately 4.5 h. Within minutes of arrival, he was completely paralysed and in respiratory arrest.

The patient required intubation soon after arrival (cold intubation without the need for paralytic agent). The endotracheal tube (ETT) was connected to an Ambu bag and he was manually ventilated (no ventilator available). There was one portable bottle of oxygen (partially filled) and a manual suction machine, both of which had to be transported from another building. A pressure immobilisation bandage was applied and the tourniquet was removed. No antivenom was available.

Due to limited resources, the patient was bag ventilated over the next 2 days with 2 L of oxygen via an oxygen concentrator. This involved a rotation of 3-h shifts for hand ventilation of the patient (one doctor, two physician assistant students, and one overseas-trained emergency nurse). His respiratory rate was titrated to his oxygen saturations. There was no access to a blood gas machine and the only blood test available was a full blood count and a whole blood clotting time (using a washed-out glass medicine ampule). The patient was given regular low-dose morphine and diazepam for sedation and comfort and commenced on intravenous antibiotics to cover for possible aspiration (based on the findings of coarse crackles on clinical examination and the unavailability of obtaining a portable chest X-ray). He was noted to have intermittent muscle fasciculations of his extremities which ceased with diazepam.

After the first night, an end tidal CO_2_ monitor was obtained. During the course of his paralysis, he had been given intravenous atropine (immediately after intubation), which markedly reduced his secretions and normalised his pupils, and neostigmine (commenced 6 h postintubation). The initial response to neostigmine was only a slight and temporary return of peripheral muscle function. These were continued at 6-h intervals. The third time (approximately 24 h post-envenomation), there was a complete reversal of his peripheral muscle paralysis (again lasting only minutes) but still not enough to maintain his oxygen saturations or reverse his ptosis. No further doses were given due to the risk of the ETT being dislodged. Bag ventilation continued and he was extubated approximately 48 h after envenomation.

His ptosis resolved over a further 6–12 h. The whole blood clotting time remained less than 10 min and urine output remained normal with no macroscopic pigmenturia. The bite site swelling reduced and there was no obvious tissue necrosis.

He recovered completely and was discharged after 5 days.

Most medically significant venomous snakes are front-fanged, of which there are three families—Atractaspidae, Elapidae, and Viperidae [1]. Elapidae are found in the tropics and are the largest and most lethal species of snake endemic to Papua New Guinea and the Solomon Islands [2]. The Elapidae family contains all venomous snakes of the Australo-Papuan and Melanesian/Pacific region [2]. The absence of the loreal scale (scale between the preocular and nasal scale) is characteristic of the Elapidae family [2].

In McCoy’s book, *Reptiles of the Solomon Islands*, only three species of Solomon Islands terrestrial elapid snakes are described [3]. These include *Loveridgelaps elapoides* (which is black and white banded), *Parapitocalamus hedigeri* (which is only in Bouganville and about 30 cm long), and the *Salomonelaps par* (which averages 75 cm in length and has a large colour and pattern variation, often deep red to dark brown, with darker transverse bands sometimes present) [3].

The snake that envenomated this patient was brought in by the family the day after he was admitted (Figure 1). It has the following distinctive features that are consistent with the *Salomonelaps par* species: approximately 90 cm in length, absent loreal scale, seven supralabial scales with the third and fourth contacting the eye, two postocular scales, and a single preocular scale [2]. The eye is considerably larger than the eye of the *Loveridgelaps elapoides* [3]. It is difficult to comment on the ventral scales due to the quality of the photo. Although this snake does not have 38 single subcaudal scales (tail was likely truncated), it does have a paired cloacal plate in contrast to the *Loveridgelaps elapoides*, which has paired subcaudals and a single cloacal plate [2]. Body colouration in this specimen consists of a series of darker vertebral blotches [2,3] in comparison to the *Loveridgelaps elapoides*, which is broadly banded black, yellow, and white [3].

Although there is documentation that the *Salomonelaps par* exists in the Solomon Islands, the effect of its envenomation is largely unknown, as there is a lack of documented cases [4,5]. O’Shea does mention that the larger species could have alarming, if not life-threatening effects [4]. Likewise, Williams describes in his book that the *Salomonelaps par* (or Solomons coral snake) could be dangerous [5].

This case confirms the suspicion that the venom from a *Salomonelaps par* produces a potentially lethal neurotoxic effect. Snake venoms are composed of different protein families, with each family containing many different toxins or toxin isoforms [1]. The venom composition of elapids was studied by Tasoulis et al., who found that about 90% of total venom composition analysed was made up of eight protein families and 75% was made up of just two protein families consisting of phospholipase A_2_s (PLA_2_s) and three-finger toxins (3FTxs) [1]. PLA_2_s commonly cause effects on the peripheral nervous system and skeletal muscles [6]. As the PLA_2_s are present in 95% of elapids [1], it would be useful to evaluate the venom of *Salomonelaps par* against commercially-available antivenoms to determine if any of these could be used clinically in treating envenomation by this snake.

This case highlights the high morbidity and mortality that can be associated with snake bites from the *Salomonelaps par* in the Solomon Islands. The World Health Organization (WHO) considers snake bites a high-priority area and is advocating for greater attention to the issue [7]. The authors suspect that there are many undiagnosed lethal snake bites in the very remote areas of the Solomon Islands. The Ministry of Health should give consideration to supporting further research and data collection in remote areas to document the number of fatalities that occur due to snake bites. They should also give consideration to supporting further research to identify appropriate antivenoms that would be effective in managing envenoming by this and other venomous snake species of the Solomon Islands. It would be beneficial to provide education for people living in very remote areas with respect to basic first aid management of snake bites. Consideration should also be given to providing formalised training for remote health staff to diagnose and treat snakebites, especially regarding supportive management of acute respiratory paralysis in the absence of mechanical ventilation as well as providing health facilities with monitoring and respiratory support equipment.

## Figures and Tables

**Figure 1:**
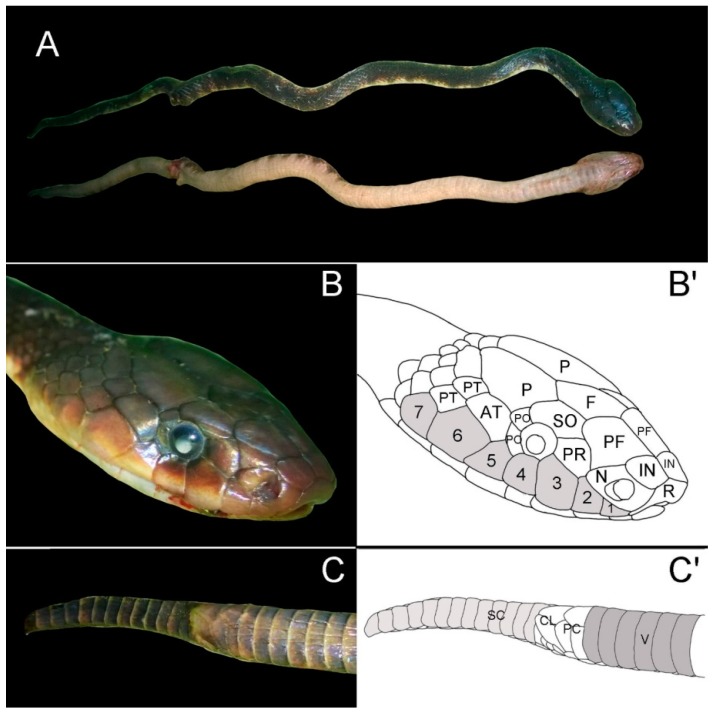
Photos and sketches of pertinent features confirming identification of *Salomonelaps par*, including: total length approximately 90 cm with a truncated tail; (**A**) dorsal and ventral body coloration and pattern consisting of dark, faintly marked patterning characteristic of *Salomonelaps par*; (**B**,**B’**) head scalation, with absent loreal scale, seven supralabial scales (numbered) with the third and fourth contacting the eye, two postocular scales (PO), a single preocular scale (PR), and a larger eye (compared to *Loveridgelaps elapoides*); and (**C**,**C’**) ventral tail scalation, with single subcaudal scales (S) (less than the specified 38 but tail appears truncated) and a paired cloacal plate (CL) in contrast to *Loveridgelaps elapoides*, which has a single cloacal plate and divided subcaudals. Figure prepared by Mark O’Shea from original images supplied by the authors.
